# Disrupting Ovarian Cancer Metastatic Colonization: Insights from Metastasis Suppressor Studies

**DOI:** 10.1155/2010/286925

**Published:** 2010-03-14

**Authors:** Shaheena Khan, Jennifer L. Taylor, Carrie W. Rinker-Schaeffer

**Affiliations:** ^1^Section of Urology, Department of Surgery, The University of Chicago, Chicago, IL 60637, USA; ^2^Committee on Cancer Biology, The University of Chicago, Chicago, IL 60637, USA; ^3^Urology Research, The Departments of Surgery, Obstetrics and Gynecology, and Medicine, The University of Chicago, Chicago, IL 60637, USA

## Abstract

Ovarian cancer affects approximately 25,000 women in the United States each year and remains one of the most lethal female malignancies. A standard approach to therapy is surgical cytoreduction, after which the remaining microscopic residual disease is treated with chemotherapy. The vast majority of patients have disease recurrence, underscoring the crucial need for approaches to control the regrowth, or colonization, of tissues after local treatment. Improved therapies require mechanistic information about the process of metastatic colonization, the final step in metastasis, in which cancer cells undergo progressive growth at secondary sites. Studies of metastasis suppressors are providing insights into events controlling metastatic colonization. This paper reviews our laboratory's approach to the identification, characterization, and functional testing of the JNKK1/MKK4 metastasis suppressor in ovarian cancer metastatic colonization. Specifically, we demonstrate that interaction of ovarian caner cells with the omental microenvironment activates JNKK1/MKK4 resulting in decreased proliferation without affecting apoptosis. The potential role of the omental microenvironment, specifically milky spot structures, is also described. It is our goal to provide this work as a usable paradigm that will enable others to study metastasis suppressors in clinical and experimental ovarian cancer metastases.

## 1. Introduction

**Figure 1:**
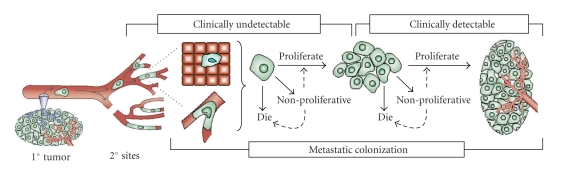
Metastatic colonization is the final step in the development of metastases. After lodging at 2° sites, cells can either remain intravascular or extravasate. To form detectable metastases, disseminated cancer cells must activate signaling cascades, enabling them to survive, enter the cell cycle, and divide. Progressive growth requires the fraction of proliferating cells to exceed the fraction of cells that are nondividing or apoptotic,(*Adapted from* [4]).

Management of metastatic ovarian cancer continues to be a critical clinical problem. Ovarian cancer affects close to 25,000 women yearly [1] and most patients have extensive metastatic disease at the time of diagnosis.Ovarian cancer metastasis is thought to result from exfoliation of tumor cells from the ovary and/or direct extension onto the peritoneal surfaces, the omentum, and the surface of organs such as the liver and bowel. A standard approach to therapy is to surgically remove surgically as much of the tumor(s) as possible, a process known as surgical cytoreduction. This technique, which leaves only microscopic residual disease, is used in conjunction with chemotherapy. Unfortunately, more than 80% of patients have cancer regrowth. These dismal statistics show the need for improved understanding of the process of *metastatic colonization*, the final step in metastasis, in which cancer cells undergo progressive growth at secondary sites [2, 3] (see Figure 1). While invasion and adhesion have been well studied, mechanisms regulating metastatic colonization are largely unknown. Studies of metastasis suppressors are providing insights into events controlling metastatic colonization [4].

Remarkably, in 2000 when our laboratory began working on metastasis suppressors in ovarian cancer, there were only a handful of papers that specifically addressed aspects of ovarian cancer metastasis. Not surprisingly, research in the molecular underpinnings of ovarian cancer metastasis continues to lag behind other cancer types. In addition to fundamental aspects of metastasis, there are promising developments in the area of therapeutic application of metastasis suppressors. Work from the laboratories of Dr. Patricia Steeg (National Cancer institute) and Dr. Dan Theodorescu (University of Virginia) demonstrates the feasibility of taking metastasis suppressors into the clinic (reviewed in [5]). The following sections describe our approach to using the JNKK1/MKK4 metastasis suppressor to dissect molecular events governing omental metastatic colonization in the SKOV3ip.1 model. It is our goal to encourage others to examine metastasis suppressors in clinical and experimental ovarian cancer metastases.

## 2. Metastasis Suppressors Can Be Used to Query the Metastatic Process and Regulate Metastatic Growth

Clinically and experimentally, tumor formation and metastasis are distinct processes. Locally growing tumors can progress without the development of metastases. This observation prompted the hypothesis that molecular processes regulating tumorigenicity and metastasis are distinguishable and could be targeted therapeutically [4]. To identify events specifically involved in metastasis regulation, our laboratory and others hypothesized that genes and their encoded proteins that specifically regulate metastasis formation could be functionally identified [4–7]. *Metastasis suppressors* are operationally defined as genes which, when ectopically expressed in metastatic cells, can inhibit the development of spontaneous overt metastases without significantly affecting primary tumor growth [4]. This definition has been extended to include *genes and their encoded proteins which specifically inhibit metastatic colonization* (i.e., experimental metastasis formation using intravenous or intraperitoneal injection) [4]. Identification of metastasis suppressors requires in vivo testing since in vitro assays generally do not model the process of metastasis.

When efforts to find metastasis suppressors were initiated, it was expected that their utility would be in predicting disease outcome; however, robust in vivo studies have showed that metastasis suppressors can control the growth of cancer cells *at metastatic sites* [4, 8]. As a result there now is evidence that metastasis suppressors can influence the interaction of disseminated cells with the microenvironment of distant organs and impair metastatic colonization. Interestingly, other investigators, working on completely different questions, also identified metastatic colonization as a rate-limiting step in metastasis formation [8, 9]. To date our laboratory and others have identified 23 *bona fide* metastasis suppressors, many of which would not have been predicted *a priori* based on their previously known function(s) [4, 5]. Determining how metastasis suppressors modulate cancer cell-microenvironmental interactions will shed light on their function in metastatic colonization, a clinically tractable therapeutic target [2, 10].

## 3. The JNKK1/MKK4 Stress-Activated Kinase Has a Novel Metastasis Suppressor Function

**Figure 2:**
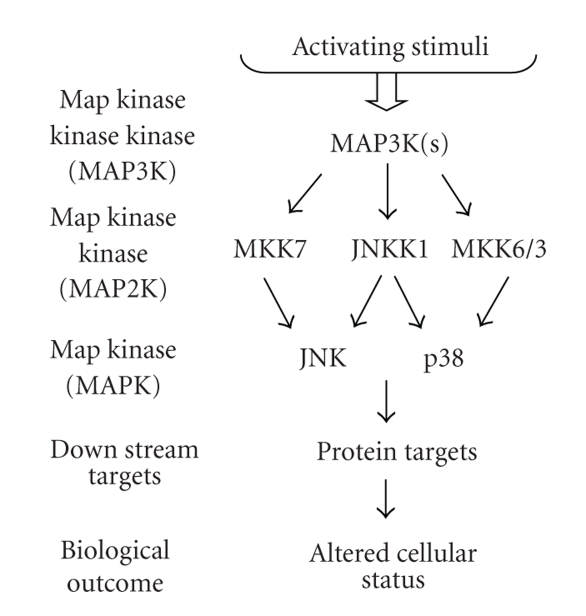
Overview of interactions in JNKK1/MKK4 signaling.

Our laboratory identified c-Jun NH2-terminal kinase (JNK) kinase 1/mitogen-activated protein kinase (MAPK) kinase 4 (JNKK1/MKK4) as a prostate cancer metastasis suppressor in 1999 [11] and subsequently as an ovarian cancer metastasis suppressor in 2002 [12]. JNKK1/MKK4 is a MAP kinase within the SAPK signaling cascade. MAP kinases occupy a central position in cell growth, differentiation, and transformation. To date, three MAP kinase modules have been well characterized: extracellular signal-regulated protein kinase (ERK), c-Jun NH2-terminal protein kinase (JNK), and p38 [13]. Each consists of a MAP3K, a MAP2K, and a MAPK. The JNK and p38 pathways are generally activated by stress stimuli. The JNK signaling cascade consists of two MAP2Ks, JNKK1, and MKK7, while the p38 signaling cascade MAP2Ks includes JNKK1, MKK3, and MKK6. JNKK1/MKK4 is a dual-specificity kinase which, in response to extracellular stimuli, can become activated and in turn can phosphorylate and activate the JNK and p38 MAPKs (Figure 2 [2–4]). In contrast, the MKK7 MAP2K can only phosphorylate JNK, while the MKK3 and MKK6 MAP2Ks can only phosphorylate p38.

Downstream targets of MAPK signaling include components of the AP-1 transcription factor complex [14]. The biological outcome of MAPK activation can depend, in part, on the transcriptional regulation of target genes. Specificity depends on factors such as cell type, cell environment, signal strength and duration, and the particular composition of the transcription factor, such as AP-1. While conventional wisdom stipulates that the JNK and p38 pathways mediate viability to stresses, increasing evidence from several model systems indicates a role for both of these MAPKs in cell cycle and consequent proliferation. For instance, reports demonstrate important functions for JNK in the G1/S transition, G2/M progression, and/or cytokinesis [15]. Similarly, p38 can activate the G2/M and spindle assembly checkpoints in mammalian cells and delay entry into mitosis or may prevent anaphase entry when the mitotic spindle is damaged [16, 17]. In sum, the biological and biochemical functions of JNKK1 were consistent with its putative role in metastasis suppression; however, there were no published studies testing its function in complex and dynamic pathological processes such as metastasis. Comprehensive in vivo studies were needed to test its role in metastasis regulation.

## 4. Testing the Ability of JNKK1/MKK4 to Suppress Ovarian Cancer Metastatic Colonization

Various studies support a role for JNKK1/MKK4 dysregulation in clinical disease [2]. In ovarian cancer, the relationship between its expression and metastasis has been particularly informative. JNKK1/MKK4 protein levels were significantly decreased in metastases as compared to normal ovarian surface epithelium [12]. Profiling studies identified high JNKK1/MKK4 expression as a significant predictor of improved response to surgical cytoreduction [18]. In vivo functional studies used SKOV3ip.1 human ovarian cancer cells, which form metastatic deposits of a serious papillary histology and produce highly reproducible numbers of metastases on the omentum, liver, and bowel [12]. After intraperitoneal injection of 1 × 10^6^ parental SKOV3ip.1 or SKOV3ip.1-vector control cells into female immunodeficient mice, the cells adhere to target organs and by 30 days post injection (dpi) animals have ∼30 metastases. SKOV3ip.1 cells have low endogenous levels of JNKK1/MKK4 but retain physiologic levels of other components of its signaling cascade [12].

**Figure 3:**
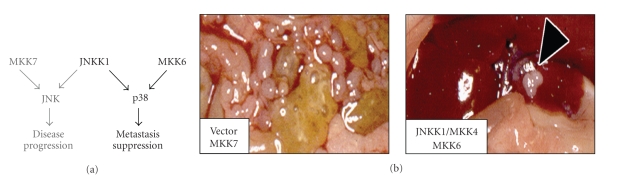
Summary of the effect of MKK7, JNKK1/MKK4, and MKK6 on SKOV3ip.1 metastasis formation. (a) Schematic of JNKK1/MKK4's signaling cascade. In vivo studies show that in SKOV3ip.1 cells, activation of p38 by ectopic expression of JNKK1/MKK4 or MKK6 causes metastasis suppression. (b) Images depicting the effect of specific proteins on metastasis formation, (*Complete primary data can be found in* [12, 19]).

Ectopic JNKK1/MKK4 decreased the number of SKOV3ip.1 metastases by 88% (*P* < .0001) and increased the animal lifespan by 70% (Wilcoxon, *P* = .0045) [12]. Its metastasis suppressor function is kinase-dependent and studies showed that selective activation of p38 by ectopic MKK6 reduced SKOV3ip.1 metastasis formation by 70% (*P* = .0082), while selective activation of JNK by ectopic MKK7 had no effect (*P* = .43) (Figure 3, 3(a) [19]). These data further defined JNKK1/MKK4's metastasis suppressor activity and prompted the question—What is the biological mechanism of JNKK1/MKK4-mediated metastasis suppression?

## 5. Determining the Biological Mechanism of JNKK1/MKK4-Mediated Metastasis Suppression

**Figure 4:**
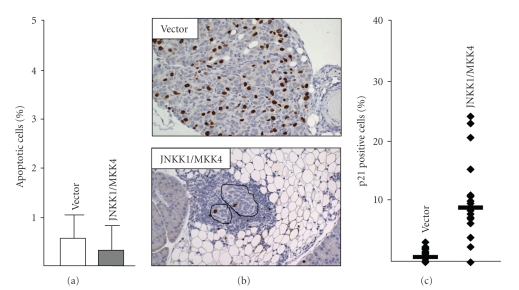
SKOV3ip.1-JNKK1/MKK4 microscopic metastases show decreased proliferation. (a) TUNEL reaction for apoptotic cells was quantitated and showed only rare positive cells. (b) Immunolabeling for BrdU in SKOV3ip1-vector and SKOV3ip.1-HA-JNKK1 microscopic metastases (outlined in black) at 14 dpi (100  × magnification). Both size and BrdU incorporation were significantly decreased in SKOV3ip.1-JNKK1/MKK4 metastases compared to SKOV3ip.1-vector metastases. (c) p21 nuclear staining was significantly decreased in SKOV3ip.1-JNKK1/MKK4 metastases compared to SKOV3ip.1-vector metastases, (*Data adapted from* [20]).

JNKK1/MKK4-mediated metastasis suppression could be due to decreased adhesion of cells, increased apoptosis of cells, or inhibition of cell proliferation. Quantitative real time PCR showed that there was not a significant difference between the numbers of vector-only and JNKK1/MKK4-expressing cells present on the omentum at 3 dpi (*P* = .06; [20]). The TUNEL reaction was used to evaluate apoptosis in SKOV3ip.1-vector or SKOV3ip.1-JNKK1/MKK4 microscopic foci. This showed rare apoptotic cells (<1%) in both groups (*P* = .43, Figure 4(a)). These data were confirmed by morphological assessment as well as immunohistochemistry (IHC) for cleaved caspase 3, which is an early marker of apoptosis [20]. To determine if SKOV3ip.1-JNKK1/MKK4 cells were deficient in proliferation, incorporation of BrdU (a marker of S-phase cells) and endogenous levels of phospho-histone H3 ((pH3), a marker of M-phase cells) were evaluated in microscopic metastases [20]. These studies showed that BrdU incorporation was decreased in SKOV3ip.1-JNKK1/MKK4 cells (Figure 4; 6% versus 19% positive cells, *P* < .0001). Similarly, pH3 staining showed decreased numbers of mitotic SKOV3ip.1-JNKK1/MKK4 cells (average of 0.7% versus 2.5% positive cells in the SKOV3ip.1-vector cells, *P* = .004) [20].

The decrease in BrdU incorporation and pH3-staining in SKOV3ip.1-HA-JNKK1/MKK4 microscopic lesions suggested that fewer cells were traversing S- and subsequently M-phase compared to controls. This prompted the examination of cell cycle inhibitory proteins, including p21 and p27, using IHC [20]. This showed a nearly 10-fold increase in p21 in SKOV3ip.1-JNKK1/MKK4 microscopic lesions in vivo as compared to controls (average 9% versus 1%, *P* < .0001, Figure 4(c)). Since only a portion of the total population of SKOV3ip.1 cells is in cell cycle at any point in time (with 19% entering S-phase in a 4-hour window), the observed increase in p21 (9% of the population) is biologically relevant [20]. The observation that JNKK1/MKK4 activation inhibits disseminated cell growth prompted us to examine the extent and duration of this suppression.

Despite the reduction in the number of SKOV3ip.1-JNKK1/MKK4 metastases at 30 dpi and extension of survival, ultimately animals succumb to metastatic disease [20]. A mathematical analysis of the rates of overt metastasis formation suggested that suppression and outgrowth of JNKK1/MKK4 cells are due to the behavior of *the population* and not selection of a subset of cells, as would occur with increased apoptosis or differential adhesion to the omentum [20, 21]. Molecular analyses showed that overt metastases still express functional JNKK1/MKK4, supporting the notion that metastasis formation was not due to selection for cells that have permanently altered their JNKK1/MKK4 signaling status [20]. Our accumulated data support a model in which binding of cells to the omentum results in the activation of JNKK1/MKK4 and induction of a cell cycle arrest [20]. In order to determine what cellular and molecular signals activate JNKK1/MKK4 and how overt metastases ultimately form, we must consider the microenvironment in which suppression is taking place. In essence we are ahead of ourselves and need to step back and consider what is known about the structure, function, and morphology of the omentum and integrate this knowledge into our current understanding of JNKK1/MKK4-mediated suppression of metastatic colonization.

## 6. Examining the Structure and Function of the Omentum and of the Omental Microenvironment

The omentum, the primary site for ovarian cancer metastases, is a fatty peritoneal fold that covers most of the abdominal organs and serves as a storage site for lipids, as a regulator of fluid exchange, and as a reservoir for immune cells [22]. Despite its importance, prevailing views of ovarian cancer metastasis formation do not consider the potentially dynamic and specialized functions that the omentum may contribute to this process. Historically, the omentum is viewed as being somewhat of an inert, black box—malignant cells attach and cancer proliferates. The implication is that ovarian cancer metastasis formation is the result of uncontrolled growth of cancer cells and not a regulated process which is in part controlled by the omental microenvironment. A review of the literature challenges the view that the omentum plays a passive role in ovarian cancer metastasis formation.

The human and murine omenta are structurally similar, being composed of both adipose-rich and translucent membranous tissues covered by a mesothelial layer [22]. Mesothelial cells share characteristics of both epithelial and mesenchymal cell types and range from flattened to cuboidal in shape, depending on the body site or state of activation [23, 24]. It is well established that omenta from a wide variety of animals, including immunodeficinet rodents, contain aggregates of immune cells known as milky spots. These were first described by von Recklinghausen in 1863 [25] and termed “milky spots” by Ranvier in 1874 [26]. In the omentum,these structures are specialized to enable mobilization of immune cells for migration into the peritoneal cavity. They may also facilitate reentry of immune cells from the peritoneum into the connective tissue (and therefore bloodstream) [18, 22–30]. Remarkably, physiologic functions of milky spots, or even their existence, have not been integrated into generally accepted models of ovarian cancer metastasis. This is a crucial oversight, as it does not consider the possibility that ovarian cancer cells may exploit a highly regulated physiologic system in order to adhere, survive, and grow into metastases.

**Figure 5:**
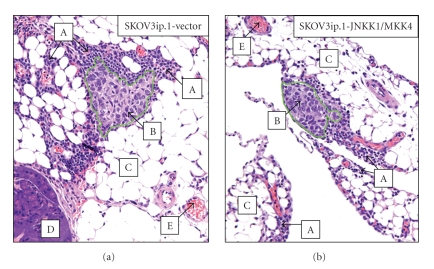
SKOV3ip.1-vector and SKOV3ip.1-JNKK1/MKK4 cells are found in association with immune cells early in the process of metastatic colonization. Histology of omental tissues harvested at 3 dpi from mice. A: immune cells; B: cancer cells (demarcated by added green border); C: adipose; D: pancreatic tissue; E: vessels (*Data adapted from* [20]).

There is a limited amount of published data that suggests that cancer cells can specifically interact with milky spot structures [31, 32]. Interestingly, in our studies, Lotan et al. found the association of SKOV3ip.1-vector and SKOV3ip.1-JNKK1/MKK4 cells with immune aggregates which we now suspect that they are milky spot structures (Figure 5 [20]). Our laboratory is currently investigating the potential role for milky spot interactions in JNKK1/MKK4-mediated suppression of metastatic colonization. We hypothesize that disseminated SKOV3ip.1 cells interact with milky spots in the omentum, and these interactions contribute to the microenvironmental context-dependent activation of JNKK1/MKK4, resulting in impaired metastatic colonization. Evidence for specific interactions of ovarian cancer cells with milky spot structures immediately identifies a target for mechanism-based studies of ovarian metastatic colonization.

## 7. Controlling Metastatic Growth by Targeting Ovarian Cancer Metastatic Colonization

There is considerable interest in controlling the growth of cancer cells at metastatic sites. Therapeutic leads may be discerned by determining why disseminated cancer cells, which have molecular modifications that should enable their growth at distant sites, often lodge at target organs and persist as undetectable, or dormant disease. Our data to date support the hypothesis that activated JNKK1/MKK4 impairs proliferation of cells early in the course of metastatic colonization. It is remarkable that few, if any, studies have been conducted that specifically examine growth control of cells during metastatic colonization. From the standpoint of translational science, the crucial yet underexplored question is how disseminated cells ultimately bypass suppression and form progressively growing metastases.

Historically, the fundamental tenets of metastasis biology dictate that acquisition of metastatic ability is the result of the “drive” of malignant cells towards growth [21]. Thus it was predicted that bypass of suppression is simply the result of mutation-selection cycles which permanently inactivate JNKK1 or members of its signaling cascade. Findings of Lotan et al. and Hickson et al. challenge this paradigm and suggest that JNKK1-mediated suppression may be due to a reversible cell cycle arrest concomitant with changes in JNKK1 activation status [20, 21]. These findings demonstrate a crucial need to reexamine important but scattered literature on population-dependent behaviors of metastatic cells, which have heretofore been refractory to mechanistic study [33–36]. This also presents an opportunity to examine the interaction of ovarian cancer cells with their microenvironment of the omentum during metastatic colonization. Given the rich literature on the bidirectional communication between cancer cells and their microenvironments, it is important that we consider microenvironmental functions and adaptations as we examine the population-dependent behaviors of cancer cells. Ultimately such studies can lay the foundation for the development of adjuvant therapies that can be used in conjunction with local therapy to delay the onset of disease recurrence, extend survival, and improve quality of life for patients with ovarian cancer.
